# Social enterprise as a model for change: mapping a global cross-disciplinary framework

**DOI:** 10.1007/s41959-022-00084-w

**Published:** 2022-11-28

**Authors:** Jamie P. Halsall, Michael Snowden, Philip Clegg, Walter Mswaka, Maureen Alderson, Denis Hyams-Ssekasi, Roopinder Oberoi, Ernest Christian Winful

**Affiliations:** 1 University of Huddersfield, Huddersfield, UK; 2 Oxford Brookes University, Oxford, UK; 3 Rollins College, Winter Park, USA; 4 City College Plymouth, Plymouth, UK; 5 University of Bolton, Bolton, UK; 6 University of Delhi, New Delhi, India; 7 Accra Technical University, Accra, Ghana

**Keywords:** Community, Social enterprise, Learning, Curriculum, Economic development, Entrepreneurship, Sustainability, Teaching and learning, Ghana, India, UK

## Abstract

Since the outbreak of COVID-19, social enterprise has experienced a renaissance. In public policy circles, entrepreneurship and innovation are perceived as economic development tools, and in many parts of the world, as catalysts for change that can have a real impact by increasing employment in communities as well as environmental challenges. At a local level, entrepreneurship and innovation enable communities to stay vibrant due to social enterprise organisations offering much-needed goods and services. Social enterprise has been acknowledged as a solution to social inequality and environmental issues in society as it develops new areas of empowerment in local communities. Central to the success of social enterprise is education, training, and the engagement of the higher education sector. Traditionally, entrepreneurship and innovation have fundamentally been entrenched within the business subject area, but have now emerged within other disciplines such as criminology, health and social care, geography, sociology, and politics. The aim of this paper is to map out a new, global, cross-disciplinary framework from a teaching and learning perspective. The authors of this paper call for global empowerment of entrepreneurship education in the higher education sector, using examples from different countries across the world, specifically Ghana, India, and the UK. This paper sets out the vital importance of entrepreneurship in teaching and learning, by showcasing what can be achieved. In this paper, the authors develop and propose a new pedagogical social enterprise model that incorporates and emphasises the ethos of ‘think globally, act locally’ in a sustainability context.

## Introduction

In light of pressing social and environmental difficulties, it is significant to recognise the political and economic dynamic forces that encourage sustainable development and to distinguish the agents that make positive and substantial changes in this direction. Currently, change makers and resilient social enterprises that can design and devise innovative solutions for multifaceted social and environmental problems are much needed (Weerawardena & Sullivan Mort, 2006). Social entrepreneurship is after all entrepreneurship with a different mission-development and sustainability. As Dees puts it, “Social entrepreneurs are one species in the genus entrepreneur” (1998, p. 3). Dees further states: “Adopting a mission to create and sustain social value: this is the core of what distinguishes social entrepreneurs from business entrepreneurs even from socially responsible businesses” (1998, p. 4). Martin and Osberg (2007, p. 34), believe that the variation between entrepreneurship and social entrepreneurship lies “in the value proposition itself”.

Today, the emerging arena of social enterprise is rapidly drawing increased attention from all sectors. It involves incredible innovation, which typifies this novel research arena, and a noticeable lack of a common framework of study. The concept of social enterprise and the apparent link between social enterprise, social change, and economic progress is an appealing prospect for scholars and policymakers alike (Oberoi, 2019). Social enterprise is branded as a multidisciplinary struggle over the epistemology of the arena that has failed to set any normative limitations around the term (Nicholls, 2010). Even though its characterisation is not yet stabilised and its boundaries remain blurred, its motivations and the aim of accomplishing both economic efficiency and social purpose are distinctive features of social enterprises (Austin et al., 2006). The blurring of sector boundaries opens up the study of social enterprise from for-profit, non-profit, and public sector perspectives. Social entrepreneurial ingenuities deny rigid classifications within organisational clusters, arranging themselves in the realm of hybridity; they cannot be categorised as belonging to any one sector. Social entrepreneurs are similarly diverse, drawing from various sectors and sources in their attempts to address social and environmental problems, which further underlines their adaptability and value.

Social enterprises enable a virtuous circle of social capital growth and environmental protection. They use social networks of support to gain access to resources and the dividends they generate are social: stronger communities, more capable of looking after themselves with the robust bonds of conviction and collaboration. Generating social capital and social benefits is at the heart of social enterprise. By connecting entrepreneurship with social change and innovation, social enterprises help communities to build up social capital, which gives them a better chance of standing on their own two feet. Social enterprise is usually used to qualify all entrepreneurial initiatives that help a social and/or environmental mission, and that return a large part of their financial surplus into their mission. Social enterprise took root within the context of financial crisis and unemployment in the 1990s, which triggered ambiguity about the future of Welfare States and their capacities to cater for novel societal needs under the neoliberal order, as well sustaining the environment. The constraints under the new post-1990s order led to the development of new relationships of interface between public and private sectors, and innovative responses to societal challenges that are workable socially, economically, and environmentally. Within this context, all forms of creativity that deals with societal wants is branded social innovation. Social entrepreneurs are mediators of constructive alteration that aim to resolve stubborn social, environmental and economic issues through novel enterprising approaches. They promote innovations and novel solutions that blend social and environmental resolution, plough proceeds into their undertakings, and are answerable for their activities.

Social enterprises are flourishing and are gradually attracting due recognition for their vast latent power and capacity for shared value creation (Oberoi et al., 2019a). Social enterprises are referred to as catalytic mediators that gradually nudge the economic system in a way that capitalist models equally concern themselves with constructive social and economic transformation, and financial markets reward these hybrid companies becoming more socially responsible. Social enterprise represents a powerful idea, an idea that is more pertinent now than ever before: social enterprises can be a medium to generate financial values while also contributing to building a fair, equitable and environmental-friendly society. As such, social entrepreneurs create pattern-breaking transformations in inequitable and unfair systems, whether through social enterprises or other social business models. They outline a swiftly growing, global actors’ assemblage that tackles social (and environmental) difficulties with entrepreneurial means. Social enterprises, which work for the connection of commercial and societal progress, are garnering attention as agents of positive change, particularly for those at the bottom of the pyramid (BoP), by pushing the restrictions in the conception and distribution of pioneering business resolutions to targeted requirements of low-income, helpless, and/or marginalised clusters (Snowden et al., 2021). With COVID-19’s epidemic disruptions and its aftermath and also effect of global warming on societies, many are inspired by more sustainable development models for the future and are openly interrogating the philosophy and structure of the global liberal order. Equally, policies and actions taken to cope with the COVID-19 pandemic have placed the global supply chains under stress and triggered a global slowdown as a result of falling economic activity; the potential for ‘economic security’ policies consistent with economic nationalism further added to the crisis (Oberoi et al., 2021). Social Enterprises are agents who perform functions and provide facilities that have formerly been seen as the sole authority of states. They are change agents, in the sense that their mission encompasses systemic solutions to structural problems rather than aid, which leave the respective institutions in place.

The study of social enterprises shows that they strategically aim to be agile and inventive, ready to act swiftly to take on the emergent concern. Because of these features, social enterprises contribute meaningfully to innovation, continuously evolving original products and facilities intended to meet societal needs. Many of these enterprises work to accomplish general modification by presenting fresh business models, shifting value chains, and triggering unexploited capacities (Oberoi et al., 2019a). Social enterprise is frequently connected to social innovation, as social entrepreneurs are probing for innovative resolutions to meet novel requirements. Jessop et al. contemplate that social innovation “is not only a descriptor for a set of practices but an emerging phenomenon, a theoretical construct and an on-going field of research within a world of social transformation” (2013, p. 2). Defourny (2001, p. 11) suggests that social enterprises can be viewed “as the expression of an innovative entrepreneurship”. As far as the classifications, associations, and theoretical dealings of social enterprise in its primary phase, it is often considered ‘a cluster’ branded by its concepts, which are hazy, overlapping, disorganised, ill-defined, and without significant theoretical underpinning (Welsch & Maltarich, 2004, p. 60).

Within the social science discipline, theoretical and empirical discourse on the theme of social entrepreneurship/social enterprise is mounting (see: Halsall et al., 2022a, b; Oberoi et al., 2019a). Entrepreneurship and education, which have promising prospects within lots of disciplines, are now taking a keen interest in the model of social enterprise. Equally significant is the influence that education has in evolving the skills that produce an entrepreneurial approach and in preparing future leaders for solving more complex, interlinked, and fast-changing problems. In the last decade and post COVID-19, social entrepreneurship is gradually making its way into the education system. In universities, the notion is beginning to gain some traction, and there are some dazzling examples in schools too. According to Katz and Antony (2010):

The expanding influence of social enterprise is reflected in the 2006 Nobel Peace Prize awarded to Muhammad Yunus, a leading promoter of microfinance and the concept of “social business;” the growth of centres for social entrepreneurship at leading business schools such as Harvard and Stanford; and media attention such as Business Week’s annual list of “America’s 25 Most Promising Social Entrepreneurs.” The Obama Administration also unveiled several initiatives to encourage the growth of social enterprise. He set up the Office of Social Innovation and Civic Participation with the goal to do business differently, by nurturing innovative community resolutions and partnership. This indicates the focus of world leaders on this model of conducting business. Even Bill Gates (a proponent of “creative capitalism”) and Pope Benedict XVI (who calls for “a profoundly new way of understanding business enterprise”) have promoted the notion of business organizations and executives making decisions that are not purely profit-driven.

In the last decade and post COVID-19, social entrepreneurship has been gradually making its way into the education system. In universities, the notion is beginning to gain some traction, and there are some dazzling examples in schools too. For example, Kirori Mal College at the University of Delhi founded the Centre for Innovation and Social Enterprise in 2020, focused exclusively on teaching and research in the domain of social enterprise. The centre was an outcome of the UKIERI project between the University of Delhi and the University of Huddersfield (2017–2021). Moreover, in a bid to strengthen innovation and bolster new start-ups, the Delhi University (DU) has set up a not-for-profit company and is working to establish another Sect. 8 firm in 2022. Defourny and Nyssens (2010) assert that social enterprise developed almost concurrently across the globe in the mid-2000s. In the US, Harvard Business School started the Social Enterprise Initiative in the 1990s, after which many colleges established support programmes for social entrepreneurs. Higher Education Institutions (HEIs) have increasingly engaged in recommending teaching for social entrepreneurship. Recently, many activities and pedagogical practices for social entrepreneurs’ training have been established (Joos & Leaman, 2014). For Oberoi et al., (2020b, p. 8):

Universities are the anchors, shapers and innovators of our communities that help to foster cultural, social and economic vitality. Learning about social enterprise gives students an opportunity to engage strongly with local businesses and communities to create all-encompassing social solutions that contribute to building stronger, more resilient, and socially engaged nations and to addressing some of the interconnected societal problems. Studying the social enterprise sector offers students thrilling professional opportunities. Combining practical and theoretical learnings help to prepare our students to be the leaders of tomorrow; mentorship is a crucial, yet often overlooked, component of social enterprise education. Opportunities for on-going support from experts in social enterprise are often limited.

But, the concept of social enterprise education is still relatively new, and education systems can be notoriously slow to change. The arrival of social enterprise on the academic scene is, however, apparent and clear. Social enterprise during COVID-19 has come with large ambitions and heroism. With unparalleled rapidity, social enterprise/entrepreneurship courses have started in top-tier business schools all over the world. Although the field of entrepreneurship scholarship is expanding, social entrepreneurship scholarship is emerging as a fresh and distinct field of its own. Entrepreneurial education alters prospects, market structures, and available resources, and new knowledge is emerging. The demand for these courses has been driven by the scholars themselves, who are enthusiastic to take courses on topics ranging from business planning and social start-ups, to entrepreneurial finance and technology management. Educators need to focus on making social entrepreneurship an attractive vocation. Universities can prepare future social entrepreneurs and provide motivational backing, forging new and lasting relationships between the public and private sectors.

The authors of this paper present an analytical discussion on why entrepreneurship and innovation are important to higher education from a teaching and learning perspective, and in an international context. This paper consists of four parts, and it begins with a brief overview of the research methodology applied in this paper. The second part of the paper provides a discussion on the connections between social enterprise and teaching and learning. The third part of the paper sets out the continued drive for solution-focused teaching and its relationship with entrepreneurship and innovation. From this, in part four of the paper the authors present a series of qualitative findings from students, academics, and social entrepreneurs and then creates and recommends a contemporary pedagogical social enterprise model that connects and underlines the ethos of ‘think globally, act locally’ in a sustainable environment. The final section concludes the paper and offers some future observations and recommendations within an enterprise higher education context.

## An overview of the methodology

The authors of this paper have undertaken ‘Action Research.’ Action research has been defined as a methodological approach that “creates knowledge based on enquiries conducted within specific and often practical contexts” (Koshy, 2005, p. 4). The authors chose action research because they want to make improvements in the current social enterprise pedagogy and, more importantly, the approach involves a three-step systematic process:

1. Action—achieved in two distinct ways. Firstly, the authors undertook a comprehensive literature review, and secondly, they held two focus group meetings. For the literature review strategy, the authors used their previous literature review practice method (see: Halsall et al., 2022a, b). Then from this, the literature review search informed the design of the focus group questions. In total there were three focus group meetings. The participants explored the contribution social enterprise makes in local, regional, national, and global contexts, and examined the skillsets required for entrepreneurship and innovation. The participants were from different stakeholder groups (i.e. academics, students, the public and private sectors, and the third sector) all of whom work in or are interested social enterprise.
2. Evaluation—undertaken after the data were collected and transcribed. Focus group transcripts were analysed and specific themes were devised.
3. Critical reflection—the final part of the process whereby the authors constructively and critically reflected on the viewpoints that were shared in the focus group meetings. As will become apparent later in this paper, a Pedagogy Social Enterprise Model (PSEM) has been devised to incorporate the changing dimensions of entrepreneurship and innovation.

## Mapping social enterprise in teaching and learning

Even though social enterprise is subject to different interpretations, there is consensus among researchers and academics that this type of business is an effective tool that can be used to ameliorate some of the most intractable socio-economic and environmental challenges that our world faces today through enterprise (Battilana & Lee, 2014; Nega & Schneider, 2014;). The social enterprise movement is also growing worldwide as an intervention to scale up innovation (Galego et al., 2018; Oberoi et al., 2022a, 2022b) as well as a mechanism to ensure economic development that is also commensurate with social justice (Mswaka et al., 2016). The growth of social enterprise, particularly in Europe (Bikse et al., 2015) and the Global North, has been characterised by an upsurge in the number of thematic areas in which this type of enterprise is working, as well as developments in policy focused on supporting this sector. Further, social enterprise has also started to attract more academic interest, due to its potential for value creation and the fact that some of the components of this burgeoning arena (Sliva & Hoefer, 2022) remain relatively unknown (Battilana & Lee, 2014).

Accordingly, HEIs across the globe, particularly in the Global North, are beginning to increase their interest in social entrepreneurship as a discipline somewhat distinct from business in the entrepreneurship education programmes and curricula. Two key reasons appear to be the impetus behind this paradigm shift. Firstly, universities worldwide are increasingly being called upon to contribute more to society beyond the delivery of teaching and learning, by producing a new generation of entrepreneurs who place the well-being of societies at the core of what they do (Ashoka, 2022). The idea behind this is to explore innovative ways of responding to and confronting the challenges that our world faces today, such as climate change, land degradation, deforestation, rising poverty, and food insecurity (Hagerdoorn et al., 2022). Secondly, there is now a greater need for higher education entrepreneurship education to produce global graduates that are also going to be responsible leaders (Hockerts, 2017). Such graduates will contribute towards efforts to transform our planet for the better through active citizenship. This is all part of what is being referred to by practitioners as the great leadership reset, which is all about creative thinking (Case Western Reserve University, 2022; Oberoi et al., 2022a, 2022b). Social entrepreneurship education curricula therefore have a critical role to play as an agent of world benefit. Despite this development, not much is known about the nature and positionality of social entrepreneurship education in teaching and learning globally. This section of our paper seeks to address this gap in knowledge by analysing social entrepreneurship pedagogy in the Global North, with examples drawn from the USA.

### The rise of social enterprise in teaching and learning: perspectives form the global north

Current literature suggests that historically, entrepreneurship education has generally been provided in business schools (Smith & Woodworth, 2012) and that social entrepreneurship has largely been subsumed under the general term of entrepreneurship. Smith and Woodworth (2012), as well as Ratten and Thukral (2020) further posit that entrepreneurship activities and experiences are multidisciplinary. As such, it is now not surprising to see entrepreneurship education in disciplines such as sport management, engineering, music, and health, as well as social sciences, in higher education institutions. What has been most surprising however, has been the rising interest in social entrepreneurship as a distinct discipline of entrepreneurship in teaching and learning that requires bespoke curricula. Smith and Woodworth (2012), as well as Worsham (2012), attribute this dimension to the desire by HEIs to involve students in developing transversal skills and competencies while at the same time ensuing that curricula take into account elements of social innovation, i.e. the practical things that students need to do to address socio-economic and environmental challenges across the globe.

Further, societies worldwide, as mentioned in preceding sections, are currently seeking innovative ways to address the myriad of challenges as well as increasing struggles and demands for racial justice. In addition, an increased consumer focus on sustainability, human rights, and social responsibility has pressured corporations across the world to do a better job of balancing profit with people and planet. What this means is that a concerted effort, involving the cooperation of business, citizens, and civil society (Cruz-Sandoval et al., 2022) and educational institutions is required to address the many socio-economic challenges that we face today. Furthermore, there is consensus that a more collaborative approach is a much more effective way to address issues and challenges embedded in the United Nations Sustainable Development Goals (SDGs). Higher Educational Institutions have responded to this call for global unity against socio-economic challenges, by putting more emphasis on recognising the power of social entrepreneurship education as an intervention that can help tackle them. In light of this, social entrepreneurship education is increasingly becoming a key feature in the teaching and learning interventions in most HEIs (Joos & Leaman, 2014) due to this greater awareness of the need to teach students about practical ways in which they can engage in active citizenship. Accordingly, key components of the emerging social entrepreneurship pedagogy must focus on skills development for the knowledge economy, awareness of global challenges, and the need for creative thinking (Garcia-Gonzalez & Ramirez-Montoya, 2021). This approach also incorporates the concept of social innovation, which Pol and Ville (2009), Galego et al. (2018), and Hagerdoorn et al. (2022) define as a process that involves a collective approach to addressing problems through sustainable solutions that can produce some form of social change. Thus, social innovation provides an overarching framework for pedagogy that seeks to enable and empower students to think of novel and creative solutions to challenges that societies in different geographical locations face, such as poverty, inequality, homelessness, health, and environmental issues (Hagerdoorn et al., 2022).

### Development of social enterprise pedagogy

Further to the issues mentioned above, there is evidence that social enterprise pedagogy is now being delivered in cognisance of broader issues of social innovation (Galego et al., 2018) and sustainability, which is defined as an intervention that meets the needs of the present without compromising the ability of future generations to meet their own needs (Brundtland, 1987). Sustainable development promotes economic and social development in ways that avoid environmental degradation, over-exploitation, and pollution (Brundtland, 1987). There seems to be an understanding that while technology exists to end poverty and deprivation, it is the will and awareness that are missing—and universities are best placed to create a new generation of students who can achieve this. A case in point, in the USA, there is now a greater emphasis in the first instance for students to have an understanding of how corporations are putting the United Nations SDGs to work, so that they become effective agents of socio-economic change. In some HEIs, courses on social entrepreneurship are designed to respond to the interest in the role that these types of businesses can take in helping to achieve the global goals in a variety of ways, as values-driven businesses (Sliva & Hoefer, 2022). Secondly, students must demonstrate how big businesses are engaging in practices and activities that go beyond profit maximisation to achieve sustainability and responsibility, through collaboration with social enterprises. To illustrate this point, there are international initiatives to support entrepreneurship between HEIs and social enterprises in Europe, The USA, and Asia, such as the Global Social Venture Competition (Hoefer & Sliva, 2016). Through this, students can obtain a greater awareness of the role social enterprises can play in such an era, and contribute towards the creation and design of radically new institutions. Thirdly, while enterprising activities in general must produce surpluses, the social entrepreneurship approach helps students to link profitability with sustainability and creation of value better (Auerswald, 2009). For example, this may entail learning about new theories of business that helps societies convert waste into wealth (upcycling and recycling), or sustainable fuels that helps in creating sustainable and resilient communities. Most importantly, an interesting dimension emerging in social entrepreneurship pedagogy in the Global North is the creation of conducive environments that help students explore their personal values and gain an understanding of what it means to be transformational leaders in the communities in which they live, and beyond. This leads us to discuss experiential and active learning practices as a key component of the new way of teaching social entrepreneurship in HEIs.

### Experiential, Active Learning and Social Enterprise Pedagogy

Experiential learning in its simplest form refers to learning from experience or ‘by doing’ (Lewis & Williams, 1994). On the other hand, active learning is a form of teaching that allows students to do something while at the same time thinking about what they are doing (Bonwell & Eison, 1991). There is no doubt that mastery of social entrepreneurship in higher education depends to a large extent on the fusion of theory and practice (Radovic et al., 2021) in exploring solutions to wicked social problems, and these two types of pedagogy are key to this. They are a component of constructivist pedagogical (CP) approaches (Bruner, 1961) and enquiry-based learning (EBL) by Kahn and O’Rourke (2005), which allow students to piece together what they are learning, as well as constructing their own learning. These approaches combined, involve the enhancement of student learning experiences through innovative, experiential, problem-based learning methods, where students scrutinise real-life projects (Wu & Martin, 2018) and devise ways of improving their impact and/or delivery of value. For example, in traditional non-entrepreneurship modules and courses such as supply chain management, students explore further how social entrepreneurship practices can help firms ensure ethical sourcing practices in their supply chains, through real-life projects and cases. Such projects provide them with opportunities to think about and suggest innovative ways to address issues in supply chains, particularly in tiers of suppliers. Students therefore learn by undertaking practical work (doing) and reflecting on the experience. In order to enhance their learning, they are also required to identify and utilise relevant concepts/theories covered in class too. For example, evaluate the integrity of supply chains and provide focused, sound, and feasible recommendations. The interesting aspect of this approach is that the multidisciplinary nature of social entrepreneurship enables tutors to devise pedagogical approaches that allow learners to explore non-economic implications of corporate actions, while sharpening their research and critical analysis skills at the same time. This also allows students to gain an awareness of what is happening around the world and, most importantly, to explore different methodologies to achieve positive societal impact. However, these approaches are typically retrospective in approach and are to be challenged in this new epoch that sees social enterprise as a key resolution strategy for social problems and challenges (Oberoi et al., 2021).

### Challenges of embedding social enterprise in teaching and learning

While the above discussions focus on the increasing consideration of social enterprise in teaching and learning, experts in higher education concur that there are still a number of challenges to be tackled, not least the availability of qualified tutors (Galego et al., 2018) and the provision of a realist curriculum (Snowden et al., 2021). These are critical in creating opportunities for entrepreneurship as well as nurturing and supporting students (Hoefer & Sliva, 2016; Galego et al., 2018). However, current research appears to suggest that there seems to be no major difference between outcomes from studying entrepreneurship generally and social entrepreneurship, and this detracts from the proposals recommended by Snowden and Halsall (2017) and Oberoi et al. (2021) in the value and importance of developing a social realist curriculum. Indeed, they go on to assert that this is vital to ensure that the social enterprise curriculum is fit for the demands of contemporary society and learning. This has, to some extent, prevented higher education from obtaining a full picture of the issues associated with the practical inclusion of social enterprise in their teaching and learning strategies. That said, educators generally agree that higher education has a profound influence on social entrepreneurship policies and intentions (Meihui, 2022), and so remains the best platform to lead on the development of social entrepreneurship through research and teaching.

## Teaching strategies utilised within social enterprise pedagogy

As posited earlier in this paper, higher education systems are notoriously slow to respond to the issues presented by societal change, and urgency is required to respond to the unprecedented global impact of the COVID-19 pandemic, and the legacy it leaves within the global community, not to mention the effect of global warming on economic lives. It is well documented that social enterprise is proposed as a valued resolution strategy to the challenges presented by the pandemic and environmental degradation (Oberoi et al., 2021; Snowden et al., 2021). However, in order to effect this change, educators must be prepared to provide an appropriate vehicle to support the development of the required knowledge, skills, and capabilities of social entrepreneurs. This section of the paper illustrated the interdependency of solution-focused learning, heutagogy and mentor-assisted learning within the dynamic context of the curriculum.

### The curriculum

Traditionally, design and delivery of the university curriculum has been a dyadic process characterised by a hierarchical relationship in which the academic determines what is taught, and how it is taught. Consequently, delivery of the social enterprise curriculum may not always respond with the required knowledge, skills and capabilities to fulfil the role of social entrepreneur. To address this, the authors of this paper propose that educators should draw upon the principal of the notion of productive knowledge. Productive knowledge is a concept presented by Snowden and Halsall (2014) who argue that in order to respond to rapid socio-economic and economic changes, curricula must demonstrate productive knowledge where the knowledge, skills and capabilities generated are those required for society to flourish and are reflected in a curriculum that is fit for purpose.

**Fig. 1:**
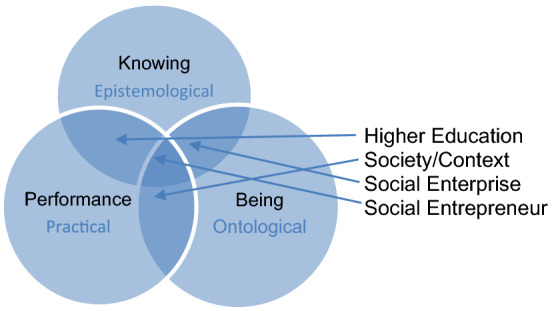
Interrelationship of curriculum components. Adapted from: Snowden and Halsall (2014)

Barnett refers to a global world that is in a state of constant change as “super complex”, dominated by global, local and regional societal change, and where competing frameworks, values and attitudes influence human understanding and needs (2011, p.6). Therefore, what constitutes learning, capability and skills in a given epoch or context may not be appropriate at another time, or in another context, and, consequently, each context, individual, group, and community is unique; at different times, their needs, skills, capabilities and knowledge must respond to their changing needs. This presents a challenge to educators, who must aspire to develop a curriculum that reflects diversity, and responds to the specific needs and demands of individuals and/or communities within any given context. Clearly, there exists within societal learning an interdependent relationship between society, learning, and knowledge, and as such, accepting that knowledge, capability, and skill acquisition is a dynamic process. Snowden and Halsall (2014) provide a conceptual model that can be used to illustrate and guide this process (see Fig. 1).

This tripartite conceptual model (see Fig. 1) illustrates the interdependent relationship between society/context, the nature of social enterprise, and higher education, and the intertwined nature of epistemology, ontology and practical features with the social entrepreneur at the centre of the learning experience.

The association and interdependence of society, knowledge and higher education is undeniable, as illustrated by Barnet (2011). However, Barnett’s assertion does not indicate what it is the learner, or in this case, what the social entrepreneur needs to fulfil the role. We identify the three core aspects as follows: (1) Epistemological—what it is that the social entrepreneur needs to know in order to execute the role, (2) Practical—that is the practical skills and capabilities to perform the role, and (3) Ontological—what they need to become, i.e. the role itself. Snowden and Halsall (2014, 2016), suggest that the importance of the ontological basis of the curriculum cannot be overstated. They assert that the ontological basis of the social entrepreneur role, that is who they are, is related to the notion of ‘self’ and forms the pillars of knowing and performing in the world, which is context specific. Therefore, in order to promote a social enterprise curriculum that is fit for purpose in preparing an aspiring social entrepreneur for the modern world, the curriculum must meet the requirements of each of the interdependent features.

While this conceptual model provides a framework for curriculum development within social enterprise, it is only the adoption of heutagogical principles alongside mentoring and solution-focused teaching and learning that it provides an opportunity to translate the curriculum into practice.

Heutagogy is described by Snowden and Halsall (2016) and Snowden (2017), as a pedagogical process that places the learner at the heart of the learning process, focusing on capability, skill, and knowledge development of the learner; distinctly, this is negotiated in the context in which they aspire to work. This process harnesses a self-determined learning approach where the leaner determines how, what, and when they learn. It is recognised by Snowden and Halsall (2016) and Oberoi et al. (2021) that learning in the twenty-first century is dynamic, and requires learners to respond proactively to new ideas and challenges, suggesting that the social entrepreneur in the Covid-era needs to be dynamic, resilient, adaptable, flexible, and responsive to change. Consequently, HEIs must develop social enterprise curricula that reflect these requirements, enabling the social entrepreneur to learn the skills, capabilities and knowledge to fulfil their role in their chosen context at an accessible time and place.

Heutagogy promotes a holistic approach to learning, and enables the learner to develop the skills and knowledge to cultivate capabilities in the subject and practice; it also encourages them to question their personal values, self, perspectives, and assumptions. This approach makes it possible for learners to easily relate their knowledge to their community’s challenges. Snowden and Halsall (2016) assert that this approach to learning is prospective and proactive—and consequently, forward looking—in approach, and knowing what and how to learn is fundamental to the learning and skills acquisition process. It is this distinct approach to learning, they suggest, that enhances capabilities, self-efficacy, resilience, and competency to work and act within a dynamic, challenging environment (Opuni et al., 2022). Furthermore, and essential for the development of the social entrepreneur, is the recognition that intuition is a fundamental feature of the learning process, drawing upon reflection and double loop and action-based learning. Heutagogy is a holistic learning approach, and to be successful, emphasis is placed upon real-world teaching that recognises the worth of self, capability, and the needs of society that focuses upon learning rather than teaching. In order to facilitate a heutagogical approach to curriculum delivery, Snowden and Halsall (2016) assert that there are two methods that are required to expedite learning: solution-focused learning and mentor-assisted learning.

### Solution-focused learning

Drawing upon Snowden et al. (2021), solution-focused learning is a teaching and learning approach that enables the leaner to become a committed, engaged social entrepreneur and to recognise that successful social enterprise delivery will require individual, societal, cultural, and institutional changes. It is a transformative learning experience that is concerned with designing solutions to challenges and issues, and by design is prospective, rather than more traditional approaches concerned with studying problems retrospectively. This approach, therefore, is especially suited to the social enterprise curriculum, as it challenges beliefs, values, and solutions, and is responsive to social injustice, oppression, inequalities, and societal change. The impact of this approach is significant, as it develops not only the cognitive domain of the learner but concurrently the affective domain of participants; it also promotes capability and competence in skill development aligned with the real-world needs of the student social entrepreneur.

Snowden and Halsall (2016) suggest that there are three sequential phases for successful solution-focused teaching and learning: (1) Assessment. The lecturer or facilitator of learning investigates and assesses the epistemological, ontological and capability skill sets of the group or individual learners. Fundamental to this process is the ability of the learner to understand the context of their study and their aspirations. This stage involves active dialogue with all collaborators and stakeholders, and furthermore requires a comprehensive assessment of need. (2) Planning and collaboration. The facilitator/teacher, in collaboration with the learner and where the relationship is viewed as an equal partnership, co-designs learning strategies that reflect the principles of how, what, where, and when as appropriate within a real-world setting. The partnership proactively constructs solutions to real-world issues, and develops capability enhancement strategies. (3) Adaptation and engagement. The learner and facilitator/teacher collaborate and engage with learning opportunities, apply newly learned knowledge, skills, and attributes within the desired entrepreneurial context (adapted from Snowden & Halsall, 2016). Teaching and learning strategies utilised successfully in this approach by the authors of this paper include: work-based learning, mentor-assisted learning, peer mentorship, case study analysis, hustings, role play and rehearsal, learning laboratories, resolution of complex solution-focused challenges, contextual and risk taking activities, data collection and utilisation exercises.

Undoubtedly mentoring in its various guises enhances learning and skill acquisition in all contexts, as suggested by Snowden and Hardy (2012), and is a process of learning facilitated by a more knowledgeable person who collaboratively facilitates personal and professional growth, and the development of a colleague or peer within a mutually beneficial relationship. The significance of the mentoring role for the development of the social entrepreneur is illustrated by Snowden et al. (2021) and reaffirmed by Oberoi et al. (2021). However, for the curriculum, Snowden and Halsall (2016) describe a mentor-assisted learning strategy that underpins and enables learning to be a scaffold within a realist, needs-led curriculum such as that afforded by a heutagogical approach. Snowden and Halsall (2016) assert that, without an embedded mentor-assisted learning strategy, the teaching and learning methods adopted fail to capitalise on the power of mentoring. The mentor, they suggest, acts as a fulcrum for the development of knowledge they provide, based on their experiences, an insight into practice and are able to provide an illustration and translation of reality within the context of learning, providing a landscape that ensures learning experience that is fit for purpose.

During the past 10 years, the authors of this article have managed several national and transnational projects related to the development and delivery of social enterprise education and training. Together, using a process of structured reflection and a training evaluation model (Kirkpatrick & Kirkpatrick, 2016), they have determined that there are three essential components of a social enterprise curriculum that is congruent with the demands of contemporary society. Firstly, the curriculum must be guided by an overall framework that recognises and responds to the relationship between the epistemological, ontological and practical (capability) features that are inseparable and determined by the interdependent relationship between society/context, higher education, and social enterprise. At the centre of this framework must be the social entrepreneur or learner at the heart of this process (see Fig. 1). However, in order to translate and reaffirm a realist curriculum that is fit for the demands of the Covid-era or society, the curriculum must adopt a heutagogical approach to teaching and learning that encourages the development of the self-determined learner who learns through a process of solution-focused teaching and learning within a mentor-assisted framework. Whilst this presents a challenge to educators, educators must embrace change, and not be afraid of learning and challenging practice when aspiring to the development of a curriculum that reflects diversity and responds to the specific needs and demands of individuals and/or communities within any given context. Failure to adopt these interdependent components will result, we suggest, in a curriculum that fails to match the aspirations of this generation.

Whilst the purpose of this section is not to propose content for the social enterprise curriculum, as discussed later in this paper, content is largely driven by business and management principles. However, previous work in this area by Oberoi et al. (2021) and Snowden et al. (2021) places emphasis upon the importance of key qualities required for the role, and present this in the form of a social entrepreneur avatar. They propose eight key personal qualities and skills required to fulfil the role of the social entrepreneur in this new epoch. These include the ability to mentor, adapt and adopt solution-focused approaches to practice, holistic in orientation and heutagogical in approach, and possess the qualities of optimism, creativity, empathy, and resilience. Each of these reflect the curriculum framework; hence, the authors of this paper propose that these components should form part of the social enterprise curriculum.

## Linking global to local: a framework

As alluded to previously in this paper, there has been a long association between the conceptual ideas of social enterprise in a local and global contexts. At an international level, social enterprise, in public policy terms, is seen as a concept that goes about solving social and economic problems. In recent years, the academic literature has noted the importance of social enterprise as a public policy tool (see Halsall et al., 2022a; Munoz et al., 2015; Oberoi et al., 2020b, 2022a, 2022b; Peredo & Chrisman, 2006; Syrett & North, 2008). As demonstrated by politicians across the world social enterprise is seen as a positive catalyst to make real impact at a community level. Moreover, Guzman (2022, p. 23) notes that in the context of the USA:

Entrepreneurship is key to the country’s economic development. High-growth entrepreneurship is a driver of innovation and increasing employment, and local entrepreneurship keeps cities and neighbourhoods vibrant, allowing access to much-needed goods and services. It serves as a solution to economic inequality and empowerment, and can produce meaningful wealth for those who succeed.

**Fig. 2:**
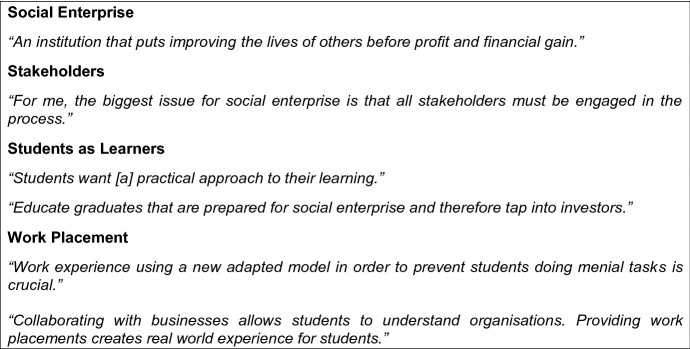
Key themed quotes from the focus group meetings

In concurrence with Guzman, quoted above, has this research discovered that social enterprise creates opportunities and solutions in the community. In the focus group meetings that were undertaken in this research project, the participants were asked to define a social enterprise, the importance of stakeholders, what students learn, and the importance of work placement. Figure 2 presents some key quotes from the participants.

**Fig. 3:**
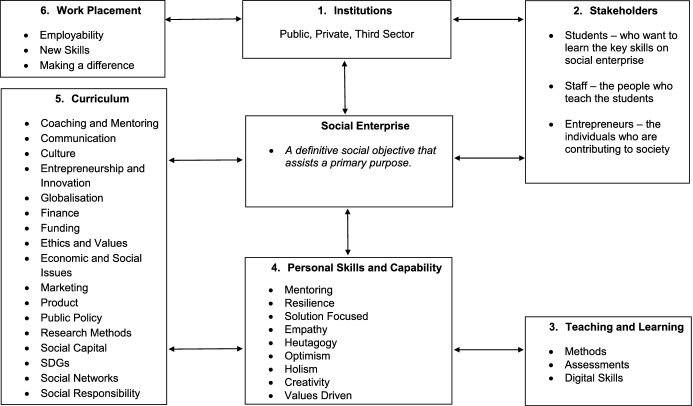
The Pedagogy Social Enterprise Model (PSEM)

As can be seen in the above themed quotes, social enterprise within an education perspective creates opportunities, challenges, and solutions. The opportunities are what social enterprise can actually do in a community setting and for the stakeholders who are involved in this process. The challenges are derived from what the students learn in an education setting. The focus here is more about real-life employability situations where social objective that assists a primary purpose forms the bases than the theoretical elements. The opportunities can be developed from the teaching and learning curriculum in terms of skills and development. Hence, drawing on the above findings the authors of this paper have developed a Pedagogy Social Enterprise Model (see Fig. 3). There are six characteristics in this model that are seen to drive social enterprise as an innovative ideology, which are:

1. Institutions—structures of rules and norms that develop social change in society. In this context, an institution is a private business, governmental or education establishment. Here, institutions are, on the whole, seen to have an important effect on citizens in society (Halsall & Powell, 2016).
2. Stakeholders—members of a particular group whose support enables an institution to function and without whom would not be able to function. Examples here are: administrators, students, teachers, and entrepreneurs.
3. Teaching and learning—a process whereby the leaner gains skills and understanding. The idea here is that the student can apply what they have learned into practice.
4. Personal skills and capability—a framework for skills and knowledge growth from a social entrepreneur development perspective. This characteristic is embedded from the authors’ earlier research (see Snowden et al., 2021).
5. Curriculum—what the leaner will cover in their course over a period of time. The curriculum is centrally driven by knowledge, practices, and critical engagement (Weller, 2019).
6. Work placement—a period where the learner has the opportunity to experience expertise in the area of employment they want to enter. As Neugebauer and Evans-Brain note, internships and placements are focused on “getting a start, establishing a track record and then adjusting from that to the path that is right” for the leaner (2016, p. 59).

Social enterprise has continued to pave its way into the local arena as a change agent, a social wealth creator, and a means to tackle the social problems that are prevalent in the community. It is clear from the study undertaken that social enterprise has positive effects on both local and regional developments. The emphasis is placed on social and economic purposes, which implies a reinvestment in the community. The study also suggests that social enterprise improves people’s lives, creates community cohesion, and promotes local economic growth. Despite the importance of social enterprise in society, students are still studying theories as opposed to acquiring practical skills. Hence, there is an urgent need to encourage students to learn by doing, and to develop an understanding of how to collaborate and engage with each other in order to address social problems. Such engagement can only take place if the key stakeholders play an active role in the transformational process, if social enterprise is embedded in the curriculum, and if opportunities to acquire skills through work-based learning, and/or structured internship programmes are offered. All in all, there is still work to be done towards the recognition of social enterprise in the local community, as well as its establishment within the academic curriculum.

## Conclusion

Social enterprise is undergoing a renaissance, and it is widely viewed as a resolution strategy to the challenges presented in contemporary society. These include social inequality and injustice, public health, and socio-environmental issues as they present in society, and distinctly, the manner in which the issues empowers communities and groups, both locally and nationally. Furthermore, social enterprise, as presented in this paper, enables communities to act as catalysts for change, promoting innovation and entrepreneurship that demonstrate a tangible impact within communities, promoting vibrancy and sustainability.

However, student learning continues to be dominated by a theory driven model, rather than acquiring the practical skills and knowledge required to fulfil their desired role. Consequently, if social entrepreneurs are to respond to societal challenge and form social enterprises that have a definitive social objective of assisting a primary purpose as a resolution strategy, there is a pressing need to encourage educators to provide students with opportunities that reflect their role and their context.

This paper emphasises the importance of education and training and proposes that in order to deliver a curriculum that is fit for purpose, a curriculum that responds to the demands of social enterprise and the development of the social entrepreneur, a reset—or at least a rethink—is required. A conceptual model (Fig. 1) has been presented that illustrates the interdependent relationship between society, the nature of social enterprise, and higher education, and the inseparable nature and influence of the epistemological, ontological and practical domains of the social entrepreneur.

Entrepreneurship education remains dominated by the business and management discipline; however, since the advent of COVID-19, it is emerging as a social science discipline that is embraced and developed within subjects such as public health, social care, geography, sociology, and politics. Undoubtedly, for a ‘new’ curriculum to develop, it must do so within a cross-disciplinary framework that is both global and heutagogical in nature as presented in Fig. 3. It is clear that the emerging social enterprise curriculum is multifaceted and complex, therefore enabling the learning experience to be dynamic and context specific to ensure that the needs of the social entrepreneur, community, and society are met by the training provider in the form of the higher education institution.

In this paper, the authors propose a new pedagogical stance: a transformative learning experience that draws together a micro- and macro-conceptual framework. The micro-model (Fig. 1) illustrates the philosophical approach that needs to be adopted, whereas the macro-model (Fig. 3) demonstrates the cross-disciplinary nature of the curriculum framework within the ethos of ‘think globally, act locally’. The authors urge educators to place greater emphasis on developing curricula that are underpinned by the models, to provide a transformative curriculum that enables the graduate of today to be prepared for the challenges of tomorrow.

It is also concluded that the combination of the micro- and macro-approach to curriculum development promotes a pedagogical paradigm shift towards heutagogy. It is asserted that this will enhance the learning experience for social enterprise students, contributing to knowledge, capability, and skill development that are congruent with the contemporary social entrepreneur that society demands.

Finally, the authors of this paper recommend further research into the state social enterprises on the various continents, which will create the platform for global social enterprise policy as well as country-specific policies. To have the societal buy-in of productive knowledge, there is a need for continuous advocacy and sensitisation of the populace on the concept of social enterprise in a global educational context.
